# Cutting into the storm: timing, benefits, and risks of ventricular tachycardia ablation across different arrhythmia substrates

**DOI:** 10.1093/ehjopen/oeaf085

**Published:** 2025-06-28

**Authors:** Frieder Braunschweig, Emmanouil Charitakis, Finn Åkerström, Nikola Drca

**Affiliations:** Department of Cardiology, Heart and Vascular Center, Karolinska University Hospital, Eugeniavägen 27, 17176 Stockholm, Sweden; Unit of Integrative Cardiovascular, Cancer- and Ageing Research (ICCA), Institution of Medicine (MedH), Karolinska Institutet, H7 Neo, 141 83 Huddinge, Sweden; Department of Cardiology, Heart and Vascular Center, Karolinska University Hospital, Eugeniavägen 27, 17176 Stockholm, Sweden; Unit of Integrative Cardiovascular, Cancer- and Ageing Research (ICCA), Institution of Medicine (MedH), Karolinska Institutet, H7 Neo, 141 83 Huddinge, Sweden; Department of Cardiology, Heart and Vascular Center, Karolinska University Hospital, Eugeniavägen 27, 17176 Stockholm, Sweden; Unit of Integrative Cardiovascular, Cancer- and Ageing Research (ICCA), Institution of Medicine (MedH), Karolinska Institutet, H7 Neo, 141 83 Huddinge, Sweden; Department of Cardiology, Heart and Vascular Center, Karolinska University Hospital, Eugeniavägen 27, 17176 Stockholm, Sweden; Unit of Integrative Cardiovascular, Cancer- and Ageing Research (ICCA), Institution of Medicine (MedH), Karolinska Institutet, H7 Neo, 141 83 Huddinge, Sweden

**This editorial refers to ‘Early vs. deferred catheter ablation of ventricular tachycardia in patients of ischemic substrate: systematic review and meta-analysis of clinical outcomes’, by A. Maan *et al.*, https://doi.org/10.1093/ehjopen/oeaf076 and ‘Cardiac function after catheter ablation of ventricular arrhythmias in patients with arrhythmogenic right ventricular cardiomyopathy’, by F. Ezzeddine *et al.,*  https://doi.org/10.1093/ehjopen/oeaf049.**

Ventricular arrhythmias (VA) remain a major cause of sudden cardiac mortality in patients with structural heart disease and contribute significantly to the global burden of cardiovascular disease.^1^ While the effectiveness of antiarrhythmic drugs remains limited, catheter ablation (CA), introduced during the late 1980s in the setting of VA, has evolved from a treatment of last-resort to an integral part of routine VA management. In particular, substrate-based ablation, the integration of high density electro-anatomical mapping with advanced imaging,^2^ and the addition of epicardial access^3^ have significantly improved procedural success, especially in patients with ischaemic heart disease. Today, VT ablation continues to progress through technological innovation and methodological development, supported by a growing body of evidence from clinical trials. Nevertheless, the efficacy, safety, and long-term outcomes of VA ablation remain under evaluation across different patient groups.

An ongoing challenge is the optimal timing of VA ablation, balancing complication risks against the potential to prevent arrhythmia recurrences, implantable cardiac defibrillator (ICD) shocks, and, ultimately, to improve survival. Furthermore, while patients with ischaemic VT account for the majority of VA ablations in clinical practice, there is a critical need to improve strategies for less common high-risk populations, such as those with arrhythmogenic right ventricular cardiomyopathy (ARVC), a condition with distinct pathophysiology and therapeutic challenges.

In this issue of the journal, two complementary articles offer important insights into the state of VT ablation. Maan *et al*.^4^ provide a systematic review and meta-analysis encompassing seven randomized controlled trials supporting the early use of VT ablation in patients with ischaemic cardiomyopathy. Outcomes of VT ablation were stratified into two groups. ‘Early’ ablation was performed prior, concomitantly or within 3 months of ICD implantation or, alternatively, within 2 months after a first appropriate ICD shock. ‘Deferred’ VT ablations were performed at least 3 months after an episode of monomorphic VT. The primary outcome was the incidence of recurrent sustained VT and secondary outcomes included the incidence of ICD shocks, VT storm, and cardiovascular mortality.

In the pooled analysis, early VT ablation was associated with a significantly lower risk of VT recurrence compared to deferred ablation with an odds ratio (OR) of 0.72 (95% CI: 0.55–0.95; *P* < 0.05) and a number needed to treat (NNT) of 4.8 to prevent one VT recurrence. Early VT ablation was also associated with significant reductions in the secondary outcomes of ICD shocks, VT storm, and cardiovascular mortality. Benefits of the early ablation strategy were most pronounced in patients with a left ventricular ejection fraction (LVEF) above 30%, who experienced a greater reduction in ICD shocks (OR 0.65, (95% CI 0.54–0.79; *P* = 0.01) compared to those with LVEF < 30%.

Taken together, the study confirms and extends previous meta-analyses on the topic^5,6^ by incorporating three recent trials (PAUSE-SCD, PARTITA, VANISH-2), and provides further evidence supporting an early or prophylactic timing of VT ablation, with significant and clinically meaningful reductions of the primary and secondary endpoints. As ICD shocks are independently associated with increased mortality and reduced quality of life due to mental health sequelae including anxiety, depression, or post-traumatic stress,^7^ the marked reduction by early ablation (NNT 6.6) has important implications to patient care. Although total mortality was not assessed, it is reasonable to expect that the observed improvements eventually translate into improved overall survival. This, however, remains to be proven in adequately powered RCTs.

Despite growing support in the literature, including studies expanding indications to non-ischaemic cardiomyopathy,^8^ early VT ablation is still not implemented as a routine clinical strategy. This hesitancy is understandable, given with the risks associated with the procedure. This was recently underscored by Eckardt *et al*.^9^ in a study analysing over 43 000 CAs, showing that VT ablation still carries the highest complication rate among all types of ablation procedures, with in-hospital mortality rates ranging from 1.3% to 1.8%. This caveat becomes particularly relevant when ablation is performed prophylactically, and individuals who might not experience a clinical benefit, nonetheless, are exposed to the procedural risks.

Hence, optimal patient selection remains a major clinical challenge. Maan *et al*. identified higher LVEF and lower LV scar burden as predictors of greater benefit from early VT ablation. However, accurately identifying candidates for preventive interventions in ischaemic cardiomyopathy is a difficult task. A recent study in over 140 000 post-myocardial infarction patients, highlighted current limitations of risk prediction modelling: neither LVEF nor other conventional variables, including MRI-based parameters, reliably distinguished individuals with low or high risk of sudden death in the setting of primary preventive ICD therapy.^10^ Meanwhile, novel pharmacologic therapies like SGLT2 inhibitors, offering dual benefits in this population by improving heart failure outcomes and reducing ventricular arrhythmia burden,^11^ add further complexity to the formulation of preventive strategies.

These observations highlight the need for a more sophisticated risk assessment. As with primary preventive ICD implantation, patient selection for prophylactic VT ablation will likely need to incorporate a wider range of predictive variables, including findings from coronary angiography and advanced imaging techniques,^12^ circulating biomarkers, genetic testing, and detailed non-invasive electrophysiological profiling. Emerging AI-driven tools may further enhance this process by enabling more precise and personalized risk stratification and treatment planning. Both registry-based studies and randomized trials are warranted to refine our understanding of optimal patient selection for early or prophylactic VT ablation.

An interesting contribution to this ongoing debate is the recent PREVENTIVE VT trial,^13^ which was not part of the present meta-analysis. PREVENTIVE VT enrolled a distinct population of patients with a myocardial scar related to chronic total occlusion of an infarct-related coronary artery (CTO) and LVEF ≤ 40% but, unlike almost all patients included in the study by Maan *et al*, no documented prior VA. Substrate ablation at the time of primary prevention ICD implantation was associated with a significant reduction of the primary outcome, a composite of appropriate ICD therapy or unplanned hospitalization for VAs (hazard ratio: 0.33; 95% CI: 0.12–0.94) compared with ICD therapy only. Although limited by its small sample size (*n* = 60), this study not only illustrates the feasibility of identifying a well-defined high-risk subgroup likely to benefit from prophylactic ablation but also challenges the traditional reliance on prior arrhythmic events as the primary determinant for ablation eligibility.

In a small but remarkable study also featured in this issue of the journal, Ezzeddine *et al.* assessed the immediate and long-term effects of VT ablation on cardiac function in 34 consecutive patients with ARVC.^14^ The authors report that 34% experienced a decline in right ventricular (RV) function, and four patients showed a reduction in LVEF. Notably, five individuals required inotropic support post-ablation. In addition, 40% of patients showed worsening tricuspid regurgitation, further contributing to haemodynamic deterioration. At one-year follow-up, recovery of RV function was rare, only one patient with an initially affected RV function improved, while two-thirds of those with impaired LV function demonstrated functional recovery.

Clinical VA management in ARVC is challenging due to a limited efficacy of antiarrhythmic drugs, the young age of most patients and the progressive nature of the disease. CA with an endocardial-epicardial approach has shown encouraging results with long-term freedom of arrhythmia recurrence in a substantial portion.^15,16^ Although clearly limited by its retrospective design and sample size, the study by Ezzeddine *et al*. raises an important clinical concern, previously not highlighted in the literature, as RV dysfunction appears to be a relatively common following VT ablation in ARVC. These findings should underscore the importance of cautious preprocedural planning through a multidisciplinary approach integrating electrophysiologists, anesthesiologists, and cardiac imaging specialists to optimize the procedural strategy and safety. Shared decision-making should include clear patient counselling about the potential risks.

Furthermore, a reasonable conclusion is that ablation procedures should be reserved for high-volume centres with expertise in epicardial ablation and access to mechanical circulatory support when needed. As the authors rightly emphasize, individualized ablation strategies that avoid unnecessary ablation of viable myocardium and consider early epicardial access may help mitigate the risk of RV failure and improve outcomes in this high-risk population. The paper also emphasizes that recurrent VT in ARVC should be considered a serious marker of disease progression potentially representing a critical turning point towards end-stage disease. Hence, these patients warrant vigilant longitudinal follow-up to ensure timely assessment for advanced therapies, including heart transplantation when appropriate.

Together, the two studies offer valuable and complementary insights into the evolving role of VT ablation across distinct patient populations. While Maan *et al.* strengthen the growing evidence in support of early intervention in patients with ischaemic cardiomyopathy, Ezzeddine *et al*. highlight the unique challenges of VT ablation in ARVC, where structural disease progression and electrical instability are even more deeply intertwined. Both studies emphasize the importance of individualized, patient tailored treatment strategies.

Catheter ablation techniques for VA continue to evolve with new energy sources like pulsed field ablation (e.g. NCT06891456) or cardiac radioablation (NCT05765175) being tested in ongoing studies. Future research should extend beyond immediate arrhythmia suppression to also address long-term outcomes, the impact on structural remodelling, quality of life, and survival. Ultimately, optimizing VT ablation requires a precision based, disease-specific approach that carefully balances procedural efficacy and safety, guided by scientific evidence and clinical judgment.

## Study acronyms

PAUSE-SCD, Preemptive Ablation of Ventricular Tachycardia in Patients with Structural Heart Disease and Subcutaneous Cardioverter-Defibrillator; PARTITA, Preemptive Ablation of Ventricular Tachycardia in Patients with Ischemic Cardiomyopathy and Recent ICD Therapy; VANISH-2, Ventricular Ablation versus Escalated Antiarrhythmic Drug Therapy in Ischemic Heart Disease—Study 2 (references to trials, see Maan *et al*.^4^); PREVENTIVE VT, Impact of Preventive Substrate Ablation of Coronary Chronic Total Occlusion on Implantable Cardioverter- Defibrillator Interventions.^13^

## Data Availability

There are no new data associated with this article.
